# Combined Medial Plate and Intramedullary Nailing for the Fixation of Extra-Articular Proximal Tibial Fractures: a Biomechanics Study

**DOI:** 10.3389/fbioe.2022.859512

**Published:** 2022-06-30

**Authors:** Yao Lu, Jiasong Zhao, Qiang Huang, Cheng Ren, Liang Sun, Qian Wang, Ming Li, Congming Zhang, Hanzhong Xue, Zhong Li, Kun Zhang, Yibo Xu, Teng Ma

**Affiliations:** ^1^ Department of Orthopaedic Surgery, Honghui Hospital, Xi’an Jiaotong University, Xi’an, China; ^2^ Bioinspired Engineering and Biomechanics Center (BEBC), School of Life Science and Technology, Xi’an Jiaotong University, Xi’an, China; ^3^ Department of International Ward (Orthopedic), Hospital of Chengdu University of Traditional Chinese Medicine, Chengdu, China

**Keywords:** biomechanical model, intramedullary nailing, malunion, osteotomy, proximal tibial fractures

## Abstract

**Purpose:** The extra-articular proximal tibial fractures continue to have high malunion rates despite development in intramedullary nailing (IMN) technology. Combined plate and IMN fixation can increase mechanical stability. The purpose of this study was to investigate combined plate and IMN for the treatment of extra-articular proximal tibial fracture using a biomechanical model.

**Methods:** A 10-mm defective osteotomy was created in the fourth-generation composite tibia to simulate extra-articular proximal tibial fractures (AO/OTA 41A2). The fractures were stabilized with IMN alone (IMN group), IMN with supplementary medial plate (M-IMN group), and IMN with supplementary lateral plate (L-IMN group). The biomechanical properties of each specimen were tested under axial compression loading, bending stress, and cyclic loading. The maximum displacement of the fragments and implant-bone construct failure was recorded.

**Results:** The maximum displacement of the M-IMN group was significantly less than either the L-IMN or IMN group in both axial compression loading and bending stress (*p* < 0.05 for both comparisons). All specimens in the three groups survived in 10,000 cyclic loading without hardware deformation. The maximum stiffness of failure was similar between the M-IMN and L-IMN groups, but the IMN group was statistically lower than either the L-IMN or the IMN group (*p* < 0.05).

**Conclusion:** The results indicated that combined medial plate and IMN fixation could effectively increase the mechanical stability of proximal tibial fractures.

## Introduction

Extra-articular proximal tibial fractures are common tibial fractures, accounting for 5%–11% of tibial fractures, which are often caused by high energy damage and accompanied by varying degrees of soft tissue damage (Court-Brown and McBirnie, 1995; Vestergaard et al., 2020). Patients always suffer from wound infection, osteomyelitis, delayed fracture union, nonunion and malunion during the long term follow ups even were treated by most sophisticated trauma surgeon (Cheng et al., 2021). Plating, external fixation, and intramedullary nailing (IMN) are common fixation methods to treat proximal tibial fractures (Tejwani et al., 2015a; Thompson et al., 2021). Open reduction and internal fixation with plates can easily cause secondary damage to soft tissues, so it has often resulted in complications such as delayed unions or nonunions, infections, and implant failures (Thompson et al., 2021). To reduce irritation and damage to soft tissue, minimally invasive plate osteosynthesis was developed and reported to have several advantages in union, alignment, and low infection rates (Gupta et al., 2016; Kim et al., 2012). The external fixation cannot achieve higher repositioning and force line maintenance requirements, and postoperative care is complex and prone to complications such as nail tract infection and deformative healing (Pairon et al., 2015). In recent years, as orthopedic surgeons have become more aware of the importance of the soft tissues and the bone blood, IMN has become a more common fixation method for proximal tibia fractures (Baker et al., 2021). Our previous meta-analysis on ten studies involving 667 patients revealed that IMN for proximal tibia fractures is associated with a shorter time of union, full weight-bearing, a low risk of infection, and fewer total complications than those for plating fixation (Ren et al., 2021). Due to the particularity of the proximal tibial anatomy, the problems of poor reduction and malunion can easily occur when fixed with the IMN (Nork et al., 2006). Currently, the additional small plate is a good solution. This study aimed to assess the biomechanical performance of combined medial plate and IMN fixation in treating proximal tibial fractures.

## Materials and Methods

### Specimens and Groups

Fifteen fourth-generation Sawbones composite tibias (left tibia, Sawbones 3401, Pacific Research Laboratories, Inc. Vashon, WA, United States), tibial nail (length 345 mm; diameter 9 mm, IRENE, Tianjin, China), and reconstruction plate (IRENE, Tianjin, China) were used for the biomechanics test. The tibial nail and plate are made of titanium alloy which have been applied clinically for fracture fixation for decades. Previous studies have demonstrated that composite tibia is similar to the tibia of a healthy adult in mechanical properties, such as axial compression, bending, and torsion stiffness (Gardner et al., 2010). Additionally, these composite models have the advantage of a lower variability for biomechanical testing (Gardner et al., 2010). The composite models were divided into three groups: group IMN, IMN fixation alone; group M-IMN, IMN with medial plate fixation; group L-IMN, IMN with lateral plate fixation.

### Fracture Model

**FIGURE 1:**
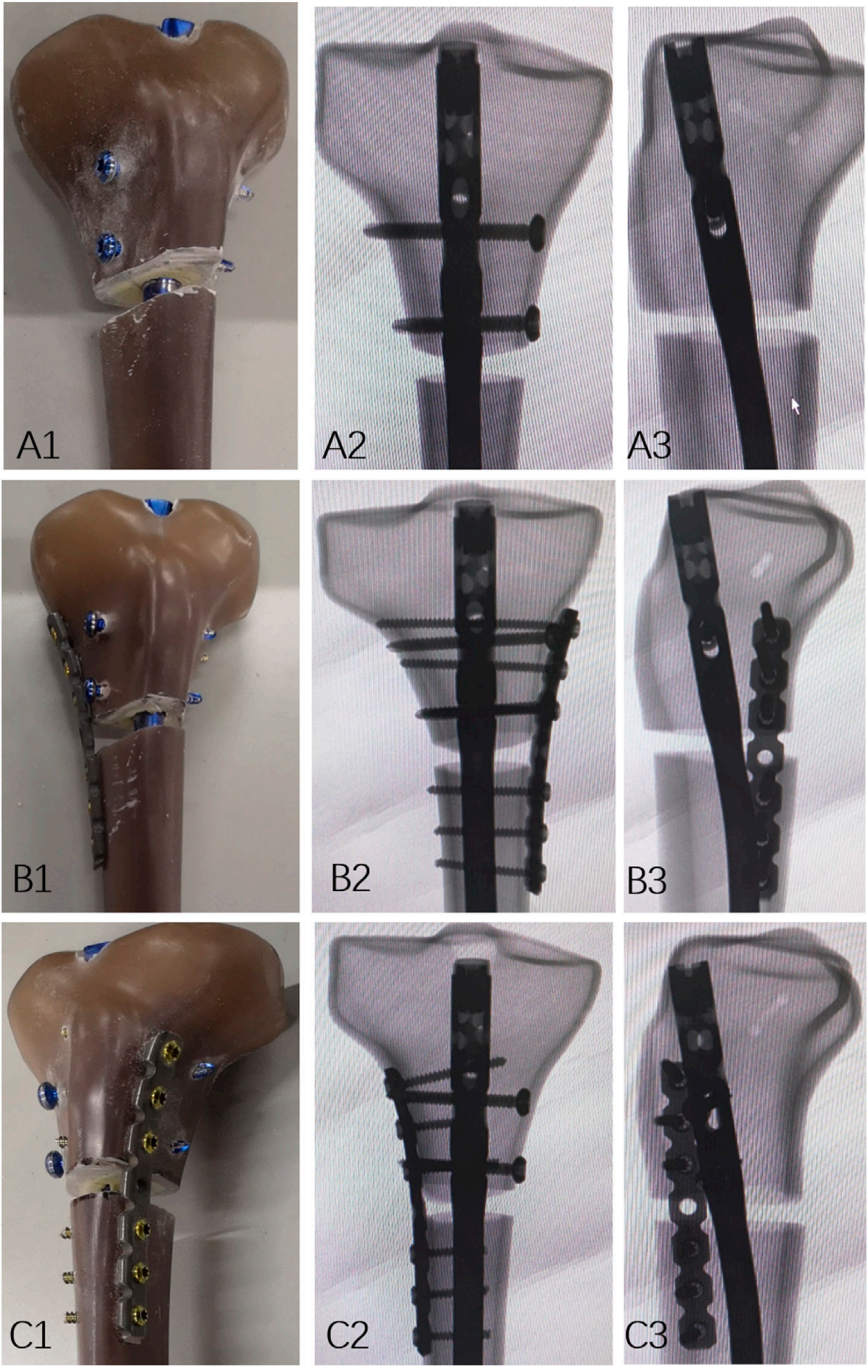
Models of the specimen. **(A1)** Photographs demonstrating IMN fixation specimens, and **(A2,3)** Fluoroscopy images. **(B1)** Photographs demonstrating IMN with medial plate fixation specimens, and **(B2,3)** fluoroscopy images. **(C1)** Photographs demonstrating IMN with lateral plate fixation specimens, and **(C2,3)** fluoroscopy images.

The tibial IMN was inserted under a standard technique in all sawbones (Lu et al., 2020). An unstable extra-articular proximal tibial fractures (OTA/AO 41A2) were created in all sawbones. All sawbones were marked at a distance of 10 mm below the second locking screw of the proximal tibia. Then, a handsaw was used to cut a 10 mm transverse defect-osteotomy (Figure1A). Five specimens received a medial 3.5 mm reconstruction plate and intramedullary nailing fixation which simulated posteromedial augmented plate fixation for extra-articular proximal tibial fractures (Figure 1B), while another five received a lateral 3.5 mm reconstruction plate and intramedullary nailing fixation which simulated anterolateral augmented plate fixation for extra-articular proximal tibial fractures (Figure 1C). Three non-locking bicortical screws were applied distally and proximally to ensure plate bone apposition, respectively. Radiographs of the three groups’ constructs are shown in Figure 1.

### Mechanical Testing

**FIGURE 2:**
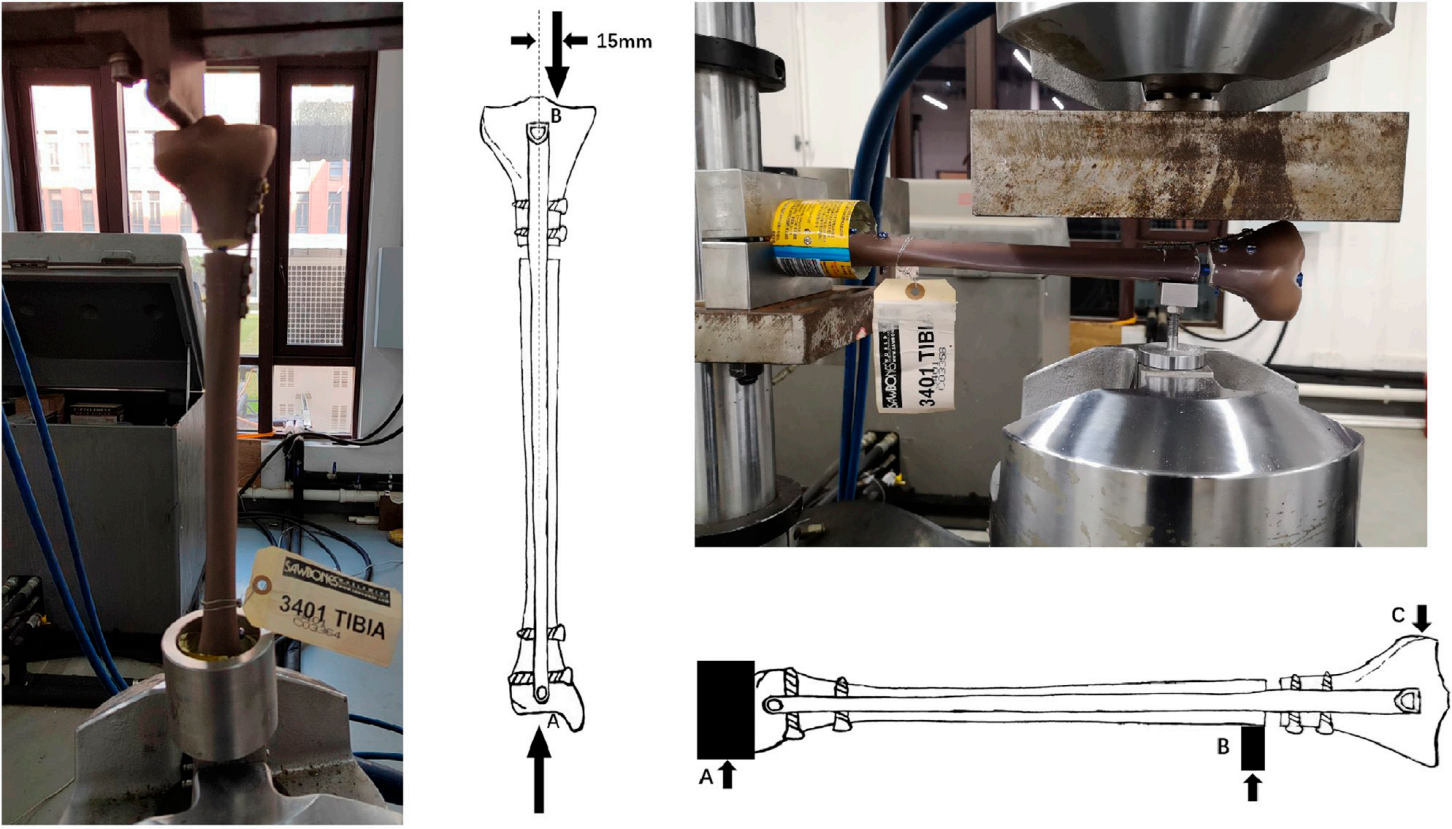
Diagrammatic sketch of axial and bending load test.

Mechanical testing was performed using a servohydraulic test system (MTS Model 810, Eden Prairie, MN, United States) (Figure 2). The test protocol of axial compression, cyclical Loading, and 3 point bending followed previous studies (Gardner et al., 2010; Zhang et al., 2021).

In the axial compression test, each specimen was performed with loading at the mechanical axis which the loading center at knee (point B) was 15 mm offset medially (Figure 2), the lower end (point A) of the specimen was fully constrained. The loads and rates in this study was based on the normal loads and rates of adults who experienced the healing stages after surgery (Hansen et al., 2007; Filardi, 2015). Each specimen was tested by initial quasi-static compression loading from 10 to 200 N at a rate of 10 N/s. Then, each specimen was tested by axial compression loading from 10 to 800 N at a rate of 10 N/s. The displacement was recorded at the fracture end corresponding to the 800 N loading force. We used the maximum displacement to evaluate the stiffness of the implants. Greater stiffness occurs with a smaller maximum displacement under the same loading (Zhang et al., 2021).

In the bending stress test, both the distal and proximal part of the specimen were fixed horizontally, the distal part (point A) was fully constrained and bending load was performed at the proximal part (point C) of the specimen, a preload of 50 N was tested at the first setting (Figure 2). Therefore, the axial compression loading (from 10 to 700 N at a rate of 10 N/s) was fixed on the medial and lateral sides at 10 mm from the proximal fracture line, respectively. The maximum lateral displacement data of the specimen was recorded.

Specimens were tested to 10,000 cycles of cyclic loading from 10 to 800 N at 2 Hz. The displacement of the fracture site between the first cycle and the last cycle was recorded. The fixed and loading methods in the cyclical loading and failure test were the same as the axial compression test. The specimens that tolerated cyclical testing were finally loaded compression loading at a rate of 10 N/s until failure. The failure of the specimen was defined as our previous study: loosened nail or bending, a bone fracture, or other heavy hardware breakages (Supplementary Figure S1); (Zhang et al., 2021).

### Statistical Analysis

Statistical analyses were computed using GraphPad 8.0 Software (La Jolla, CA, United States). The Shapiro-Wilk test was used to determine whether the continuous variables were normally distributed. Data satisfying normality were reported as means and standard deviations. A non-parametric test or Student’s *t*-test was used to compare the differences between two groups. One-way ANOVA was used to compare independent measurements. *p*-values < 0.05 indicated a statistically significant difference.

## Results

**FIGURE 3:**
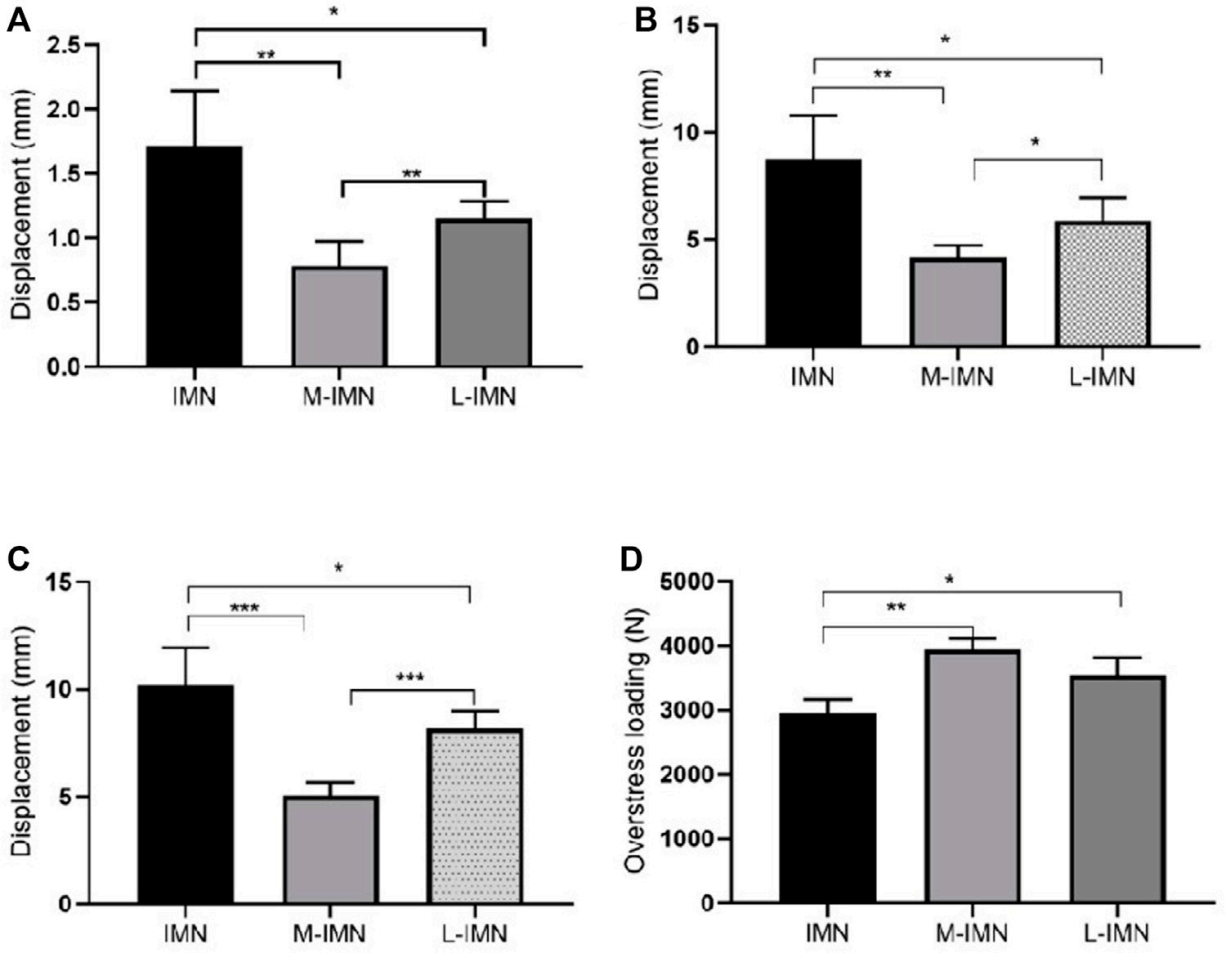
Biomechanical performance in different models. The maximum displacement of IMN, M-IMN, and L-IMN group under axial compression **(A)**, medial side **(B)**, and lateral side **(C)** bending stress, respectively **(D)** The maximum loading of failure was in three groups. * <0.05; ** <0.01; *** <0.001.

In the axial compression stiffness test at 800 N, the maximum displacement was summarized as follows: 1.72 ± 0.43 mm, 0.78 ± 0.19 mm, and 1.16 ± 0.13 mm, for group IMN, group M-IMN, and group L-IMN, respectively. Compared with the IMN group, the mean maximum displacement of M-IMN and L-IMN groups decreased by 54.65%, 32.56%, respectively. The 3-level (group IMN, group M-IMN, and group L-IMN) one-way ANOVA revealed that the maximum displacement was significantly different for the groups (*p* < 0.05 for both comparisons) (Figure 3A).

In the 3-point bending stress test on the medial side at 700 N, the maximum displacement was summarized as follows: 8.72 ± 2.06 mm, 4.15 ± 0.59 mm, and 5.88 ± 1.09 mm, for group IMN, group M-IMN, and group L-IMN, respectively (Figure 3B). Compared with the IMN group, the mean maximum displacement of M-IMN and L-IMN groups decreased by 52.41% and 32.57%, respectively. The maximum displacement was summarized as follows: 10.19 ± 1.75 mm, 5.04 ± 0.61 mm, and 8.18 ± 0.82 mm, for group IMN, group M-IMN, and group L-IMN, respectively on the lateral side (Figure 3C). Compared with the IMN group, the mean maximum displacement of M-IMN and L-IMN groups decreased by 50.54% and 19.73%, respectively. The 3-level (group IMN, group M-IMN, and group L-IMN) one-way ANOVA revealed that the maximum displacement was significantly different for the groups at medial side and lateral side bending stress (*p* < 0.05 for both comparisons) (Figures 3B,C).

**FIGURE 4:**
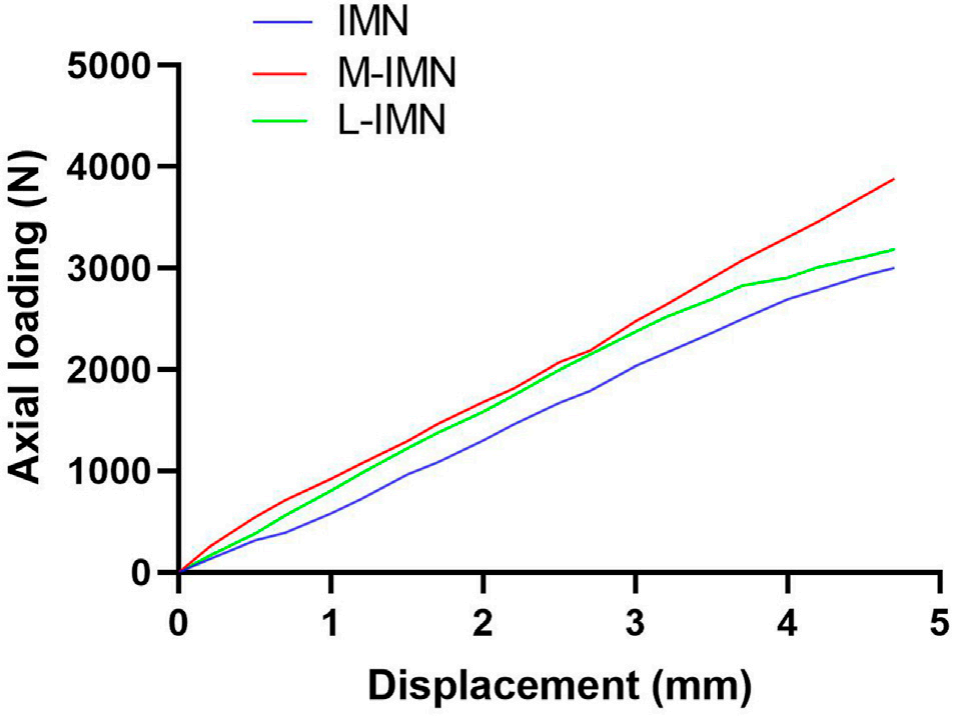
Axial compression loading curves in different models.

All specimens in the three groups survived in cyclic loading. The mean maximum loading failure was 2943 ± 225.5 N, 3940 ± 170.9 N, and 3538 ± 275.3 N for IMN, M-IMN, and L-IMN, respectively. The maximum loading failure was similar between group M-IMN and L-IMN, but group IMN was the lowest (*p* < 0.05) (Figure 3D). The loading curves of three groups were shown in Figure 4.

## Discussion

Both plate and IMN can be successfully used to treat extra-articular proximal tibial fractures. Although typing of fractures can guide decision-making, implant selection is often based on the preferences of orthopedic surgeons. Our previous study revealed that IMN for proximal tibia fractures is associated with a shorter time of union, full weight-bearing, a low risk of infection, and fewer total complications than plating fixation (Ren et al., 2021). Moreover, some authors reported an alternative fixation option for intramedullary fracture fixation by elastic nailing recently, according to previous study when synthesis devices are too stiff the bone is not properly stimulated and healing is delayed, therefore the numerical elastic self-locking nail maybe another better fixation option (Putame et al., 2020). However, when dealing with the extra-articular proximal tibial fractures, even titanium alloy nailing resulted in delayed union and nonunion due to lack of fixation stability of the tibial proximal fragment, trauma surgeons had to choose augmented options such as poller screws or plates to maintain the stability of the fracture. Although the incidence of proximal tibial fractures is only 11% of all tibial fractures, the incidence of malunion is up to 16.7%–84% and is accompanied by a loss of proximal fracture fixation (Krieg, 2003; Padubidri et al., 2021). To reduce or avoid the malunion of proximal tibial fractures after IMN, Dunbar et al. (2005) first proposed that the 3.5 mm system plate was used to fix proximal tibial fractures, and then IMN was used for fixation. The 33 patients all achieved the effect of anatomical reduction. However, biomechanical studies of an additional plate with IMN are currently lacking. Here, we evaluated the stability of combining IMN and additional plate fixation in treating proximal tibial fractures in this composite tibia gap osteotomy model.

The biomechanical properties of healthy adult human bones depend on different factors. Consistent structural properties of the bones are important. Evaluation of the structural properties outcomes of fourth-generation composite tibia models and the healthy adult human bones in a previous biomechanical study showed that composite specimens had significantly lower variability than cadaveric models for all loading regimens (Gardner et al., 2010). Another previous biomechanical investigation also reported that the variability of the fourth-generation composite specimens was less than 6% for all cases, which was much less than that of the natural human bones (28%) (Heiner, 2008). The fourth-generation composite tibia specimens were applied in our investigation, and the different groups were compared.

There is great interest in identifying alternative implants with more excellent biomechanical stability to treat proximal tibial fractures. Medial locking plate, lateral locking plate, double plate (Medial-lateral plate), suprapatellar and infrapatellar approaches of IMN, and supplementation of the plate with IMN are feasible techniques for proximal tibial fractures (Tejwani et al., 2015b; Jindal et al., 2021; Thompson et al., 2021). Lee et al. (2014) used a biomechanical study to compare the mechanical properties of different internal fixation methods in treating proximal tibial fractures. They found that IMN was significantly higher than the single or double plates in terms of ultimate strength. In a biomechanical test, Matthias et al. (Hansen et al., 2007) investigated five devices (IMN, conventional double-plate osteosynthesis, external fixator, unreamed tibial nail with a T-stabilization-plate, and the less invasive stabilization system) for the treatment of extra-articular proximal tibial fractures. They found that the IMN could theoretically provide similar mechanical stability to the double-plate in axial loading.

Additionally, IMN could provide higher stability in axial loading than external fixator or the less invasive stabilization system. Similarly, a biomechanical study comparing the fatigue strength of the IMN construct with a double-plate construct in comminuted extra-articular proximal tibial fractures in fresh frozen cadaveric tibias indicated that IMN could provide the same fatigue performance as the double-locked plates (Kandemir et al., 2017). In the current study, the mean maximum displacement in the M-IMN group is less than that in IMN or L-IMN group in axial loading. In other words, M-IMN can provide higher mechanical stability in axial loading than IMN or L-IMN.

With the development of IMN technology, the incidence of malunion remains high in the treatment of proximal tibial fractures. Padubidri et al. (2021) reported that the rate of malunion was 16.7% (5/30) with extra-articular proximal tibial fractures after IMN. A recent meta-analysis and systematic review indicated that the rate of malunion was 28.1% (18/64) with proximal tibial fractures after IMN (Jindal et al., 2021). Mild varus and valgus malunion can lead to poor alignment of joint, increase the rate of reoperation and the treatment cost, and eventually cause the occurrence of traumatic arthritis, affecting the prognostic function and social function of patients (Saragaglia et al., 2020). Several studies reported that supplementary plate fixation adds stability to tibial fractures and reduces the rate of malunion (Dunbar et al., 2005; Yoon et al., 2015). So, we evaluated the lateral displacement of three fixation methods (IMN, L-IMN. and M-IMN) for proximal tibial fractures by a 3-point bending stress test. Our results showed that the mean maximum displacement in the M-IMN group is less than that in IMN or L-IMN group on both medial and lateral side loading.

Immediate weight-bearing is one of the advantages of IMN for tibial shaft fractures (Gross et al., 2016). If extra-articular proximal tibial fractures or distal tibial fractures are shown to have the same safety as tibial shaft fractures, the choice of implants, operative and postoperative protocols may be changed. Hadeed et al. (2021) used a biomechanical model to evaluate immediate post-fixation stability of the distal tibial fractures with IMN. They reported that immediate weight-bearing is safe for well-reduced simple fractures. Our previous study found that combining the plate with IMN for proximal tibial fractures allows patients to be weight-bearing early (Guo et al., 2020). The present study showed that all specimens in the three groups survived in cyclic loading, the L-IMN group provided stronger ultimate strength than the IMN group, and the M-IMN group proved to be the strongest. This biomechanical data supply a better understanding of safety evidence for full weight-bearing on proximal tibial fractures after supplementing the plate with IMN.

There are several limitations in the present biomechanical study. 1) The experimental model used in this study is the four-generation composite tibia. Although it can simulate the mechanical properties of normal human bone, there is still a certain gap compared with the specific mechanical environment containing soft tissue *in vivo*. Fresh or frozen cadaveric tibia is much closer to normal human bone in biomechanical properties. 2) Torsional stress experiment was not performed in this study due to defects in the test machinery. 3) Medial and lateral bending stress were tested for this study. There is a lack of front and posterior bending stress tests. 4) The fracture model used in this experiment is artificial. The fractured end is neat and simple, which can not truly reflect the actual situation of clinical fracture. The type of fracture is relatively homogeneous and does not reflect well the type of implant suitable. The data obtained from the study have limitations for clinical application. 5) The cyclic loading of 10,000 cycles are not enough to establish fatigue strength. In our further work we may be try to accomplish more than 5 million cycles. Moreover, the loading set up has two full constrain at both ends. The results seems to be in un-physiologic loads. During our future work of the further study on the biomechanical properties of the fixation options above, the finite element models could be established to overcome the foresaid limitations and the relevant analysis results may prove the validity of the mechanical test.

## Conclusion

The proximal tibial fractures continue to have high malunion rates despite development in IMN technology. In a composite model of proximal tibial fractures, combined medial plate and IMN fixation significantly increase stability. Whether these small biomechanical advantages can be transferred into clinical practice has to be evaluated in further clinical studies.

## Data Availability

The original contributions presented in the study are included in the article/Supplementary Material, further inquiries can be directed to the corresponding authors.
