# Supplementary figures and images for: Combined Medial Plate and Intramedullary Nailing for the Fixation of Extra-Articular Proximal Tibial Fractures: a Biomechanics Study
Source: Front Bioeng Biotechnol. 2022 Jun 30;10:859512. doi: 10.3389/fbioe.2022.859512 (PMC9280979; doi:10.3389/fbioe.2022.859512)

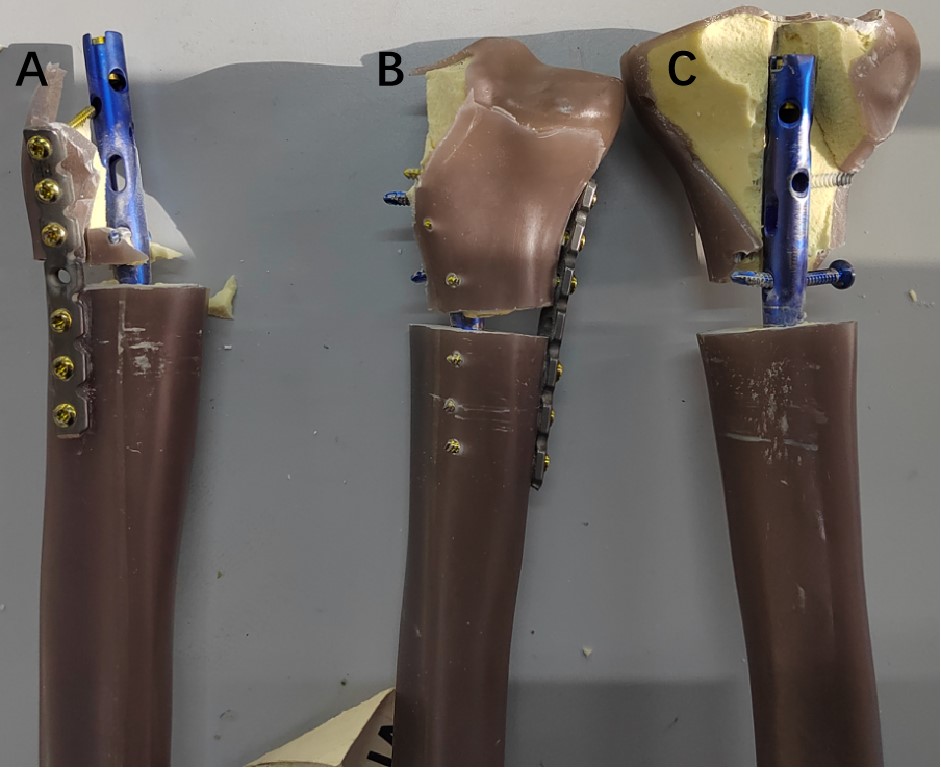


Fig.S1 A photograph of broken sample. A,M-IMN. B,L-IMN. C,IMN.

Supplement: Supplementary file 2 [file DataSheet1.doc]
